# Ischemia and reperfusion injury to mitochondria and cardiac function in donation after circulatory death hearts- an experimental study

**DOI:** 10.1371/journal.pone.0243504

**Published:** 2020-12-28

**Authors:** Oluwatoyin Akande, Qun Chen, Stefano Toldo, Edward J. Lesnefsky, Mohammed Quader

**Affiliations:** 1 Department of Surgery, Virginia Commonwealth University, Richmond, VA, United States of America; 2 Division of Cardiology, Department of Medicine, Virginia Commonwealth University, Richmond, VA, United States of America; 3 Medical Service, McGuire Veterans Administration Medical Center, Richmond, VA, United States of America; 4 Department of Surgery, McGuire Veterans Administration Medical Center, Richmond, VA, United States of America; Indiana University School of Medicine, UNITED STATES

## Abstract

The ultimate treatment for patients with end-stage heart failure is heart transplantation. The number of donor hearts which are primarily procured from donation after brain death (DBD) donors is limited, but donation after circulatory death (DCD) donor hearts can increase the heart donor pool. However, ischemia and reperfusion injuries associated with the DCD process causes myocardial damage, limiting the use of DCD hearts in transplantation. Addressing this problem is critical in the exploration of DCD hearts as suitable donor hearts for transplantation. In this study, rat hearts were procured following the control beating-heart donor (CBD) or DCD donation process. Changes in mitochondria and cardiac function from DCD hearts subjected to 25 or 35 minutes of ischemia followed by 60 minutes of reperfusion were compared to CBD hearts. Following ischemia, rates of oxidative phosphorylation and calcium retention capacity were progressively impaired in DCD hearts compared to CBD hearts. Reperfusion caused additional mitochondrial dysfunction in DCD hearts. Developed pressure, inotropy and lusitropy, were significantly reduced in DCD hearts compared to CBD hearts. We, therefore, suggest that interventional strategies targeted before the onset of ischemia and at reperfusion could protect mitochondria, thus potentially making DCD hearts suitable for heart transplantation.

## Introduction

Heart Failure (HF) affects 6 million adults in the United States [1]. Patients with end-stage HF require heart transplantation (HTx) to improve survival [2]. Presently, most transplantable hearts come from donation after brain death (DBD) donors [3]. However, the availability of these donors is limited [3, 4]. Donation after circulatory death (DCD) donors can expand the heart donor pool. In DCD donors, organs are procured following death pronouncement based on “irreversible cessation of circulatory and respiratory functions” [3]. While DCD donation has lessened the shortage of donors of solid organs such as kidneys, lungs, livers [5], DCD hearts are not routinely used for HTx mainly because of the inherent ischemic insult resulting from the DCD process. In contrast to DBD donors that have intact cardiorespiratory function before procurement, DCD heart donors suffer from warm ischemia, which is the interval from the withdrawal of mechanical ventilation to initiation of coronary perfusion with cold organ preservation solution. In addition, during the DCD process, the heart experiences a rapid surge of catecholamines and volume overload that also contribute to the myocardial damage [6].

Mitochondria are key targets of myocardial injury during ischemia and reperfusion [7]. Mitochondrial function is known to be impaired in DCD organs such as kidney and liver [8, 9]. Perfusion with hypothermic oxygenated buffer improved mitochondrial function in DCD pig livers [9]. The aim of our current study is to characterize heart mitochondrial function after circulatory death with and without reperfusion. Once we accurately quantify the damage to mitochondria with the DCD process, future studies can be designed to test the effectiveness of interventions to mitigate the mitochondrial damage in DCD hearts. The two cardiac mitochondrial subpopulations, subsarcolemmal mitochondria (SSM), located beneath the plasma membrane and interfibrillar mitochondria (IFM), located between the myofibrils, differ in functionality. They are also known to be differentially affected by ischemia [7, 10]. Mitochondria generate adenosine triphosphate (ATP) through oxidative phosphorylation (OXPHOS) via the electron transport chain (ETC) located in the mitochondrial inner membrane [7]. Ischemia and reperfusion damage the mitochondrial respiratory chain that leads to increased reactive oxygen species (ROS) generation and intercellular calcium overload. Oxidative stress and calcium overload increase cardiac injury by inducing the opening of mitochondrial permeability transition pores (MPTP), a non-selective pore located in the mitochondrial inner membrane. The opening of MPTP increases the permeability of the mitochondrial inner membrane that causes depolarization of inner mitochondrial membrane potential and a decrease in ATP synthesis. MPTP opening also increases in response to cytosolic calcium overload, leading to respiratory chain uncoupling and membrane potential collapse [11–14]. MPTP opening also increases the permeability of the outer mitochondrial membrane that leads to a release of mitochondrial proteins including cytochrome *c* into cytosol which triggers programmed cell death [11]. Thus, MPTP opening is one of the leading causes of cardiac injury during ischemia and reperfusion. Most of the knowledge on the series of events and on the extent of damage to the mitochondria in the ischemic or reperfused hearts comes from *ex-vivo* ischemia studies [15–18]. A recent study showed that the DCD process (*in vivo* ischemia) leads to mitochondrial dysfunction similar to the *ex vivo* studies but with distinct differences [19]. In this study we will address the changes in mitochondrial and cardiac function from DCD hearts subjected to different periods of *in vivo* ischemia and *in vitro* reperfusion.

## Materials and methods

All experiments were conducted per the ‘Guide for the care and use of laboratory animals’ published by the National Institutes of Health [20]. The Animal Care and Use Committees of the McGuire VA Medical Center and Virginia Commonwealth University approved the present study.

### Study design (Fig 1)

Male Sprague-Dawley rats were anesthetized with sodium pentobarbital (100 mg/kg intraperitoneally) and ventilated while monitoring heart rhythm with EKG. Heparin (1000 U/kg intraperitoneally) for anticoagulation and vecuronium (0.5 mg/ml intramuscular) to paralyze skeletal muscles were administered. The average heart rate for CBD hearts was 395 bpm (mean ± SEM, 395 ± 11, n = 8). CBD hearts were procured without stopping the ventilator. The DCD set-up was induced by stopping ventilation and observing asystole, followed by a preset ischemia time of 25 or 35 minutes before procuring hearts. The average heart rate for DCD hearts before stopping ventilation was 409bpm (mean ± SEM, 409 ± 9, n = 8). The rats were divided into 6 groups to quantify the differences in mitochondrial function with varying durations of *in vivo* ischemia with and without reperfusion: CBD without reperfusion (n = 5), CBD with 60 min of reperfusion (n = 8), DCD 25 min of ischemia (n = 5), DCD 35 min of ischemia (n = 5), DCD 25 min ischemia + 10 min reperfusion (n = 8), and DCD 25 min ischemia +60 min reperfusion (n = 8). Rat hearts were perfused on a Langendorff setup with modified Krebs-Henseleit (KH) buffer (115 mM NaCl, 4.0 mM KCl, 2.0 mM CaCl_2_, 26 mM NaHCO_3_, 1.1 mM MgSO_4_, 0.9 mM KH_2_PO_4_, and 5.5 mM glucose) oxygenated with 95% O_2_/5% CO_2_, at 37°C perfused at a steady 72 mmHg pressure. After 10 min of reperfusion (RP), a latex balloon tip catheter was inserted into the left ventricle to monitor left ventricle function. Left ventricle developed pressure (LVDP), myocardial contractility (+dP/dt) and myocardial relaxation (-dP/dt) were measured and calculated using Labchart software (ADInstruments Inc., Colorado Springs, CO). Rate pressure product (RPP = heart rate X LVDP) was used to account for cardiac function variability with heart rate (HR).

**Fig 1:**
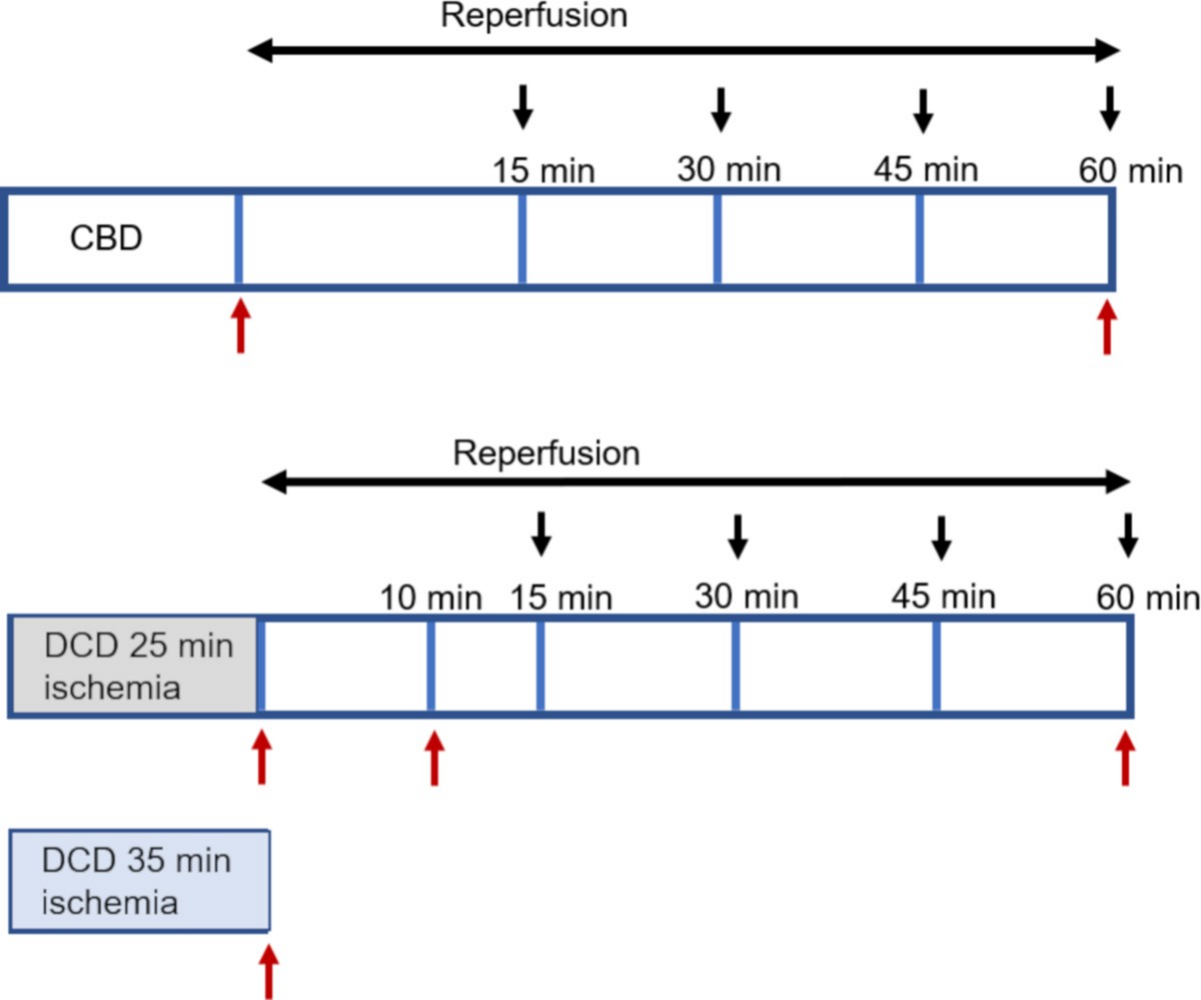
Experimental design. Horizontal black arrows represent the reperfusion timeline for donation after brain death (CBD) or donation after circulatory death (DCD) hearts. Black arrows pointing downwards represent time intervals where the cardiac function was measured. Red arrows pointing upwards represent time points where rat hearts were collected for mitochondria isolation to study oxidative phosphorylation and calcium retention capacity.

### Isolation of SSM and IFM

Hearts were collected for mitochondrial isolation with or without reperfusion, according to our previously published protocol [21]. Briefly, heart tissue was first placed into cold (4°C) buffer A [100 mM KCl, 50 mM 3-(*N*-morpholino) propanesulfonic acid (MOPS), 1 mM EGTA, 5 mM MgSO4·7 H2O, and 1 mM ATP, pH 7.4], then was minced in buffer B (Buffer A + 0.2% bovine serum albumin) and homogenized with a polytron tissue processor (Brinkman Instruments, Westbury, NY) for 2.5 seconds at the 10,000 rpm. The homogenate was centrifuged at 500 x *g* for 10 min. The supernatant was collected and centrifuged at 3000 x *g* for 10 min to sediment SSM. The pellet from polytron homogenate was re-suspended in buffer A, homogenized and incubated with trypsin [15] (5 mg/g wet weight) for 10 min at 4°C. The homogenate was centrifuged at 500 x *g s*for 10 min and the supernatant was further centrifuged at 3000 x *g* for 10 min to sediment IFM. The SSM and IFM were washed twice with buffer B, and then re-suspended in KME buffer (100 mM KCl, 50 mM MOPS, and 0.5 mM EGTA). Mitochondrial protein content was measured by the Lowry method, using bovine serum album as a standard.

### Mitochondrial oxidative phosphorylation

Oxygen consumption by mitochondria was measured using a Clark-type oxygen electrode at 30°C as previously described [21]. Briefly, mitochondria were incubated in 80mM KCl, 50mM MOPS,1mM EGTA,5mM KH_2_PO_4_, and 1mg of defatted, dialyzed bovine serum albumin/ml, pH 7.4. Glutamate (20 mM, complex I substrate), succinate (20 mM) plus 7.5 μM rotenone (complex II substrate), and TMPD (N,N,N’,N’ tetramethyl p-phenylenediamine, 1 mM)-ascorbate (10 mM, complex IV substrate) + rotenone were used. Glutamate reduces NAD^+^ to NADH that provides electrons to complex I, whereas succinate reduces FAD^2+^ to FADH_2_ that donates electrons to complex II. TMPD supplies electrons to cytochrome *c* and then cytochrome oxidase (complex IV). Maximal ADP-stimulated respiration (2 mM ADP) was also determined.

### Determination of calcium retention capacity

Calcium retention capacity (CRC) was used to assess the sensitivity of MPTP opening in the isolated mitochondria. Mitochondria (400 μg/ml) were incubated in buffer containing 150 mM sucrose, 50 mM KCl, 2 mM KH_2_PO_4_, 5 mM succinate in 20 mM Tris/HCl, pH 7.4. MPTP was triggered by the administration of sequential pulses of calcium (20 nmol) until the calcium was released from mitochondria. Extra-mitochondrial Ca^2+^ concentration was recorded with 0.5 μM Calcium Green-5N and fluorescence monitored with excitation and emission wavelengths set at 500 and 530 nm, respectively, using LS-5 fluorimeter (Perkin Elmer, Waltham, MA).

### Lactate dehydrogenase release assay

Lactate dehydrogenase (LDH) content was measured from coronary effluent collected during reperfusion. Briefly, coronary effluents were diluted and incubated with potassium phosphate (100mM, pH 7.4) for 5 minutes at 37°C in the presence of NADH. Pyruvate was added to start the reaction. LDH content was quantified by measuring the rate of NADH consumption (absorbance change at 340nm) using an Agilent spectrophotometer (Hewlett-Packard model 8453, Waldbronn, Germany) [15].

### Detection of H_2_O_2_ production

The rate of H_2_O_2_ production in mitochondria was determined using the oxidation of the fluorogenic indicator amplex-red in the presence of horseradish peroxidase [22]. The concentrations of horseradish peroxidase and amplex-red in the incubation medium were 0.1 unit/ml and 50 μM, respectively. Fluorescence was recorded with 530nm excitation and 590nm emission wavelengths. H_2_O_2_ production was initiated in mitochondria using glutamate (10mM), or succinate (5mM). Rotenone (2.4μM) was added to the incubation medium to inhibit the activity of complex I.

### Immunoblotting

Cytosolic cytochrome *c* contents were detected from the cytosolic fraction obtained during mitochondrial isolation. Briefly, 5ug of cytosolic protein were fractionated by SDS-PAGE and transferred to a polyvinylidene difluoride membrane using a transfer apparatus according to the manufacturer’s protocols (Bio-Rad). After incubation with 5% nonfat milk in TBST (Tris Buffered Saline, 1.0% Tween 20) for 60 min, the membranes were incubated with antibodies against cytochrome *c* (mouse monoclonal, Trevigen 6370-MC-100, 1:1000 dilution) or GAPDH (rabbit polyclonal, Cell signaling 5174S, 1:1000 dilution) at 4°C overnight. Membranes were washed three times for 10 min and incubated with horseradish peroxidase-conjugated anti-mouse or anti-rabbit secondary antibodies (1: 10,000 dilution) for 1 h. Blots were washed with TBST three times and developed with the ECL system (Amersham Biosciences) according to the manufacturer’s protocols.

### Infarct size measurement

The triphenyl tetrazolium chloride (TTC) staining method as described by Fishbein MC et al., *Am heart J*; 101:593–600, 1981 was employed to measure infarct size Hearts perfused for 90 minutes were sliced transversally at a thickness of 1 mm and immersed in 1% TTC solution for 20 min at 37°C followed by storing the heart slices in 10% formaldehyde overnight. Once stained, the heart slices were individually weighed and scanned. The scanned images were processed using image tool software to determine the total infarct size in relation to the heart weight.

### Statistical analysis

Values are expressed as the mean and ± standard error of the mean (SEM). Comparisons between multiple groups (≥ three groups) were performed using a one-way analysis of variance (ANOVA) followed by Student-Newman-Keuls analysis for multiple groups when data passed the normality test. Comparisons between two groups were performed with a two-tailed non-paired, student t-test. A p-value less than 0.05 from either one-way ANOVA or student t-test was considered significant.

## Results

### Effect of DCD induced ischemia on oxidative phosphorylation (OXPHOS)

**Fig 2:**
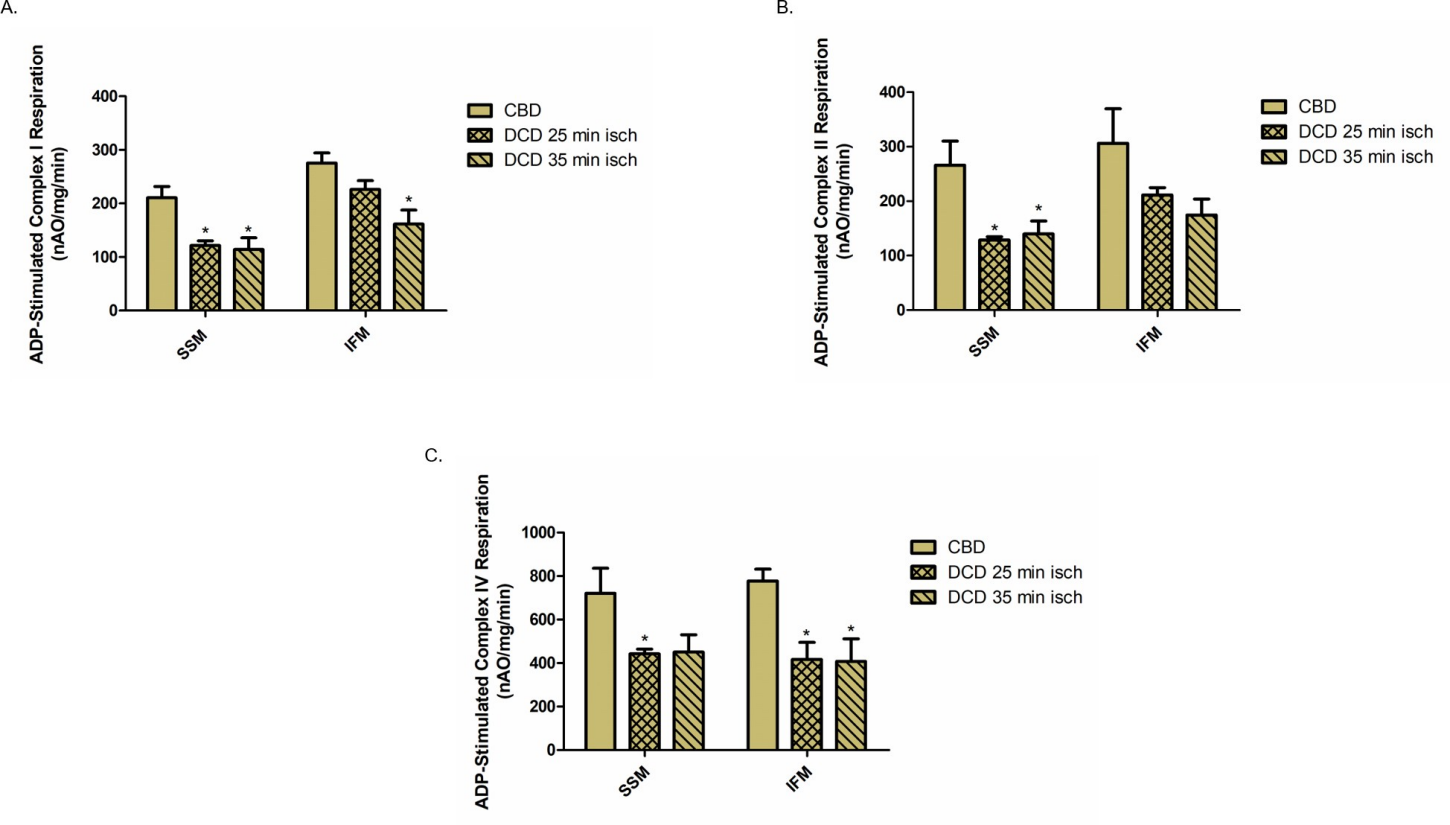
Oxidative phosphorylation in mitochondria from DCD and CBD hearts. Oxygen consumption using glutamate, succinate, or TMPD-ascorbate as complex I, II and IV substrates respectively, were measured in SSM and IFM. (A.) ADP-stimulated glutamate oxidation (complex I) from 0.25mg of SSM and IFM from CBD and DCD hearts. (B.) ADP-stimulated succinate oxidation (complex II) from 0.25mg of SSM and IFM from CBD and DCD hearts. (C.) ADP-stimulated TMPD oxidation (complex IV) from 0.125mg of SSM and IFM from CBD and DCD hearts. n = 5 in each group. Data are expressed as means ±SEM. *p<0.05 vs CBD, using one-way ANOVA.

To determine the effect of DCD induced ischemia on OXPHOS, we measured the rate of oxygen consumption in the two mitochondrial populations, SSM and IFM, from hearts subjected to 25 minutes or 35 minutes of ischemia (Fig 1). Compared to CBD hearts, the rate of ADP-stimulated OXPHOS was decreased by 42% and 46% in SSM from DCD hearts with 25 minutes and 35 minutes of ischemia respectively when glutamate was used as complex I substrate (Fig 2A). However, in IFM, 25 minutes and 35 minutes of ischemia only led to a 13% and 41% decrease in OXPHOS respectively with complex I substrate (Fig 2A). Compared to CBD hearts, OXPHOS was also decreased in SSM (52% with 25 minutes of ischemia and 47% with 35 minutes of ischemia) when succinate was used as complex II substrate with the addition of rotenone to block reverse electron flow (Fig 2B). IFM from DCD hearts also exhibited 31% (25 min ischemia) and 43% (35 min ischemia) decrease in OXPHOS using succinate as complex II substrate (Fig 2B). Additionally, SSM from DCD hearts exhibited a significant 39% reduction in OXPHOS with 25 minutes of ischemia and 37% reduction with 35 minutes of ischemia compared to the CBD heart when TMPD+ascorbate was used as complex IV substrate (Fig 2C). There was a 46% and 47% reduction in OXPHOS in IFM with 25 and 35 minutes of ischemia oxidizing complex IV substrates (Fig 2C). These results suggest that SSM and IFM exhibit differential sensitivity to *in vivo* ischemia-induced mitochondrial damage with SSM exhibiting greater sensitivity to ischemic injury. Also, 35 minutes of ischemia did not further decrease the OXPHOS rate compared to 25 minutes of ischemia.

### Effect of reperfusion on OXPHOS in CBD and DCD hearts

**Fig 3:**
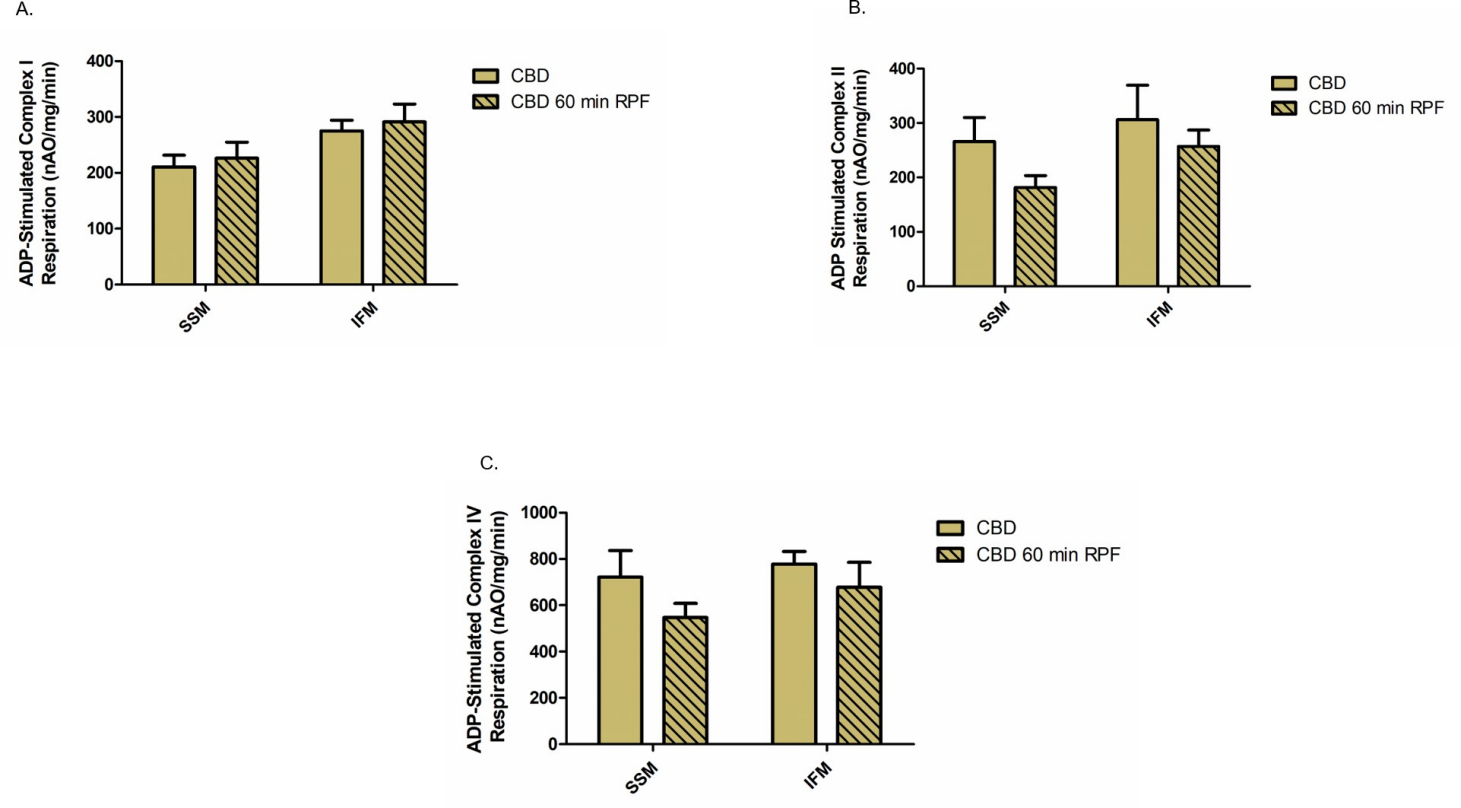
Effect of reperfusion on oxidative phosphorylation in mitochondria from CBD hearts. Oxidative phosphorylation was measured in subsarcolemmal (SSM) or interfibrillar mitochondria (IFM) from CBD hearts with no reperfusion (n = 5) or with 60 minutes of reperfusion (RPF), n = 8. Oxygen consumption using glutamate, succinate, or TMPD-ascorbate as complex I, II and IV substrates respectively, were measured in SSM and IFM (A-C). Data are expressed as mean ±SEM.

**Fig 4:**
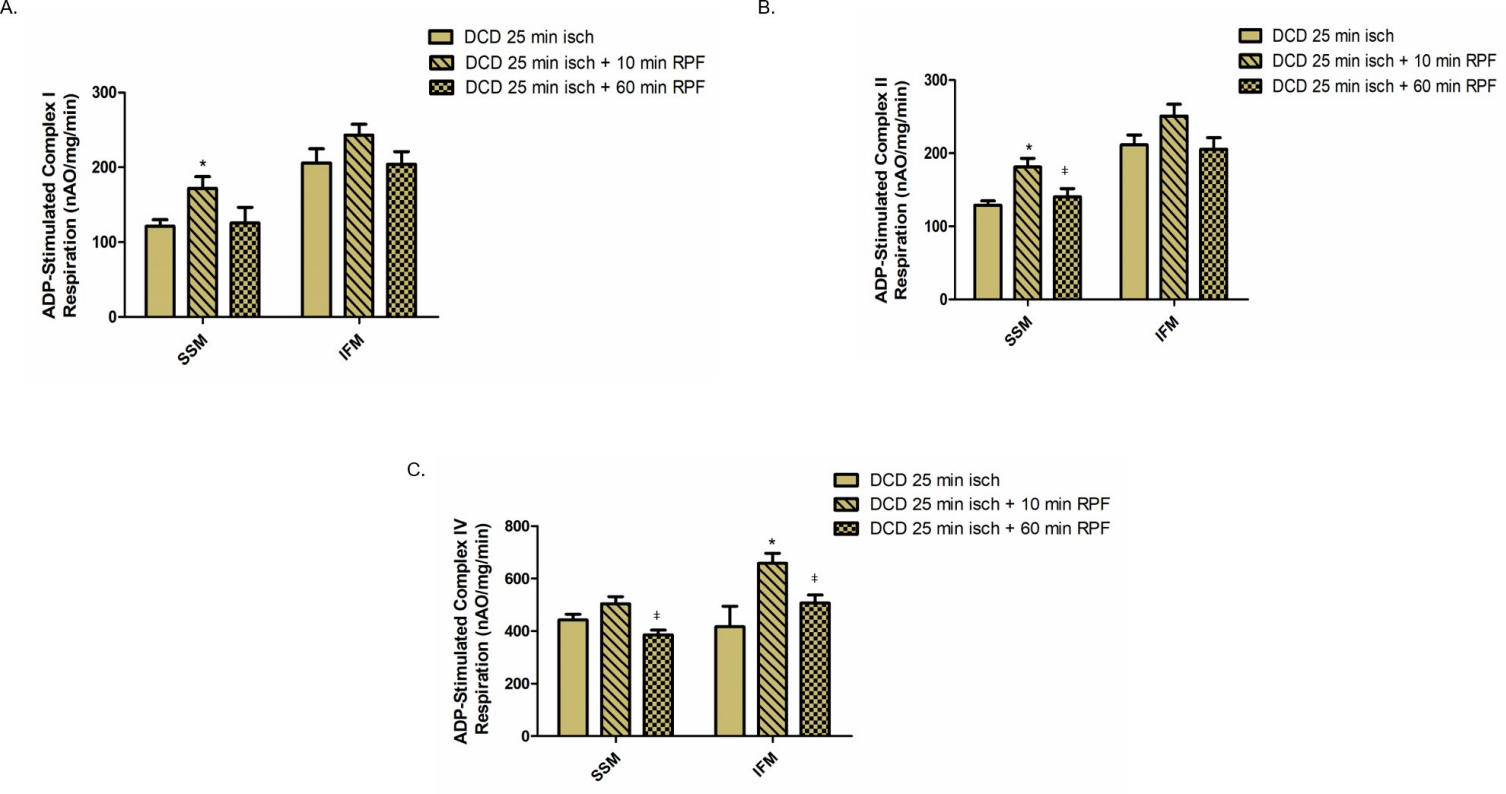
Effect of reperfusion on oxidative phosphorylation in mitochondria from DCD hearts. Oxidative phosphorylation was measured in subsarcolemmal (SSM) or interfibrillar mitochondria (IFM) from DCD hearts with no reperfusion (n = 5), with 10 minutes of reperfusion (n = 8) or with 60 minutes of reperfusion (n = 8). Oxygen consumption using glutamate, succinate, or TMPD-ascorbate as complexes I, II and IV substrates respectively, were measured in SSM and IFM (A-C). Data are expressed as mean ± SEM. *p<0.05 vs DCD hearts with no reperfusion group; ^ǂ^p<0.05 vs DCD hearts with 10 min reperfusion group, using one-way ANOVA.

Since DCD-induced ischemia caused OXPHOS dysfunction, we determined if reperfusion caused an additional decrease in OXPHOS. We first studied if KH buffer perfusion for 60 minutes alone leads to alteration of OXPHOS in mitochondria from the control beating (CBD) hearts. There were no significant differences in the OXPHOS function of both SSM and IFM from CBD hearts with reperfusion, oxidizing complex I, II or IV substrates (Fig 3A–3C). Next, we assessed in DCD heart mitochondria the effect of different periods of reperfusion (10 minutes vs. 60 minutes) on the OXPHOS function. Since 35 minutes of ischemia did not worsen OXPHOS in DCD hearts versus 25 minutes of ischemia, we examined the effect of reperfusion on OXPHOS in DCD hearts subjected to 25 minutes of ischemia. Following 10 min of reperfusion, OXPHOS was slightly improved in SSM compared to DCD hearts without reperfusion using complex I, II, and IV substrates. However, OXPHOS was decreased in mitochondria following 60 minutes of reperfusion compared to 10 minutes of reperfusion. Interestingly, there were no significant differences in OXPHOS in SSM or IFM from DCD hearts with 60 minutes of reperfusion compared to DCD hearts with no reperfusion (Fig 4A–4C). These results suggest that OXPHOS is slightly improved with the initial period of reperfusion but decreased as reperfusion continued.

### Effect of ischemia and reperfusion on calcium retention capacity (CRC)

**Fig 5:**
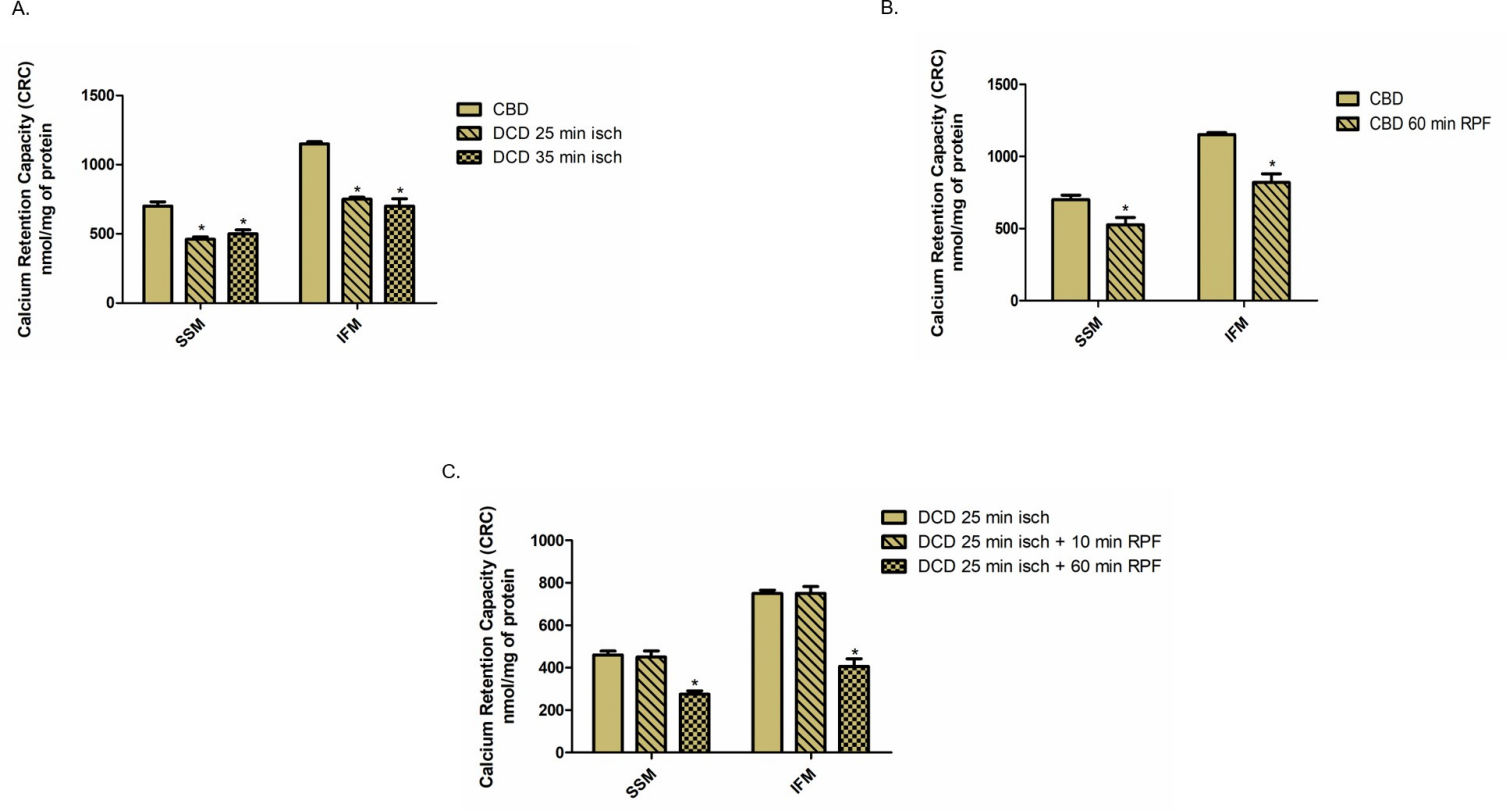
Calcium retention capacity in mitochondria from CBD and DCD hearts with and without reperfusion. (A). Subsarcolemmal (SSM) or interfibrillar mitochondria (IFM) were isolated from CBD hearts (n = 5) or from DCD hearts with 25 or 35 minutes of ischemia (n = 5, each). Pulses of calcium were added at 1minute intervals to 0.4mg protein of SSM or IFM incubated with calcium retention capacity (CRC) buffer and calcium green. *p<0.05 vs CBD group, using one-way ANOVA. (B.) SSM and IFM were isolated from CBD hearts with no reperfusion or with 60 minutes of reperfusion (n = 8). CRC was measured as described above. *p<0.05 vs CBD group with no reperfusion, using two tailed non paired t-test. (C.) SSM and IFM were isolated from DCD hearts with no reperfusion (n = 5), with10 or 60 minutes of reperfusion (n = 8, each). CRC was measured as described above. Data are expressed as mean ±SEM. * p<0.05 vs DCD with no reperfusion group; ^ǂ^p<0.05 vs DCD with 10 minutes of reperfusion group, using one -way ANOVA.

Since ischemia and reperfusion lead to the opening of the non-selective mitochondrial permeability transition pores (MPTP), we examined CRC, a measurement of susceptibility to MPTP opening in response to ischemia and reperfusion. Compared to the CBD hearts, CRC was decreased by 34% and 30% in SSM and by 34% and 39% in IFM from DCD hearts with 25 minutes and 35 minutes of ischemia, respectively (Fig 5A). Compared to non-perfused CBD hearts, buffer perfusion alone led to 25% and 29% decrease in CRC in SSM and IFM, respectively (Fig 5B). In DCD hearts with 25 minutes of ischemia, 10 minutes of reperfusion did not lead to a significant alteration in CRC in either SSM or IFM (Fig 5C). However, 60 minutes of reperfusion resulted in a 40% and 46% decrease in CRC in SSM and IFM respectively (Fig 5C). These results indicate that the DCD process sensitizes to MPTP opening, which is further worsened during prolonged reperfusion. In addition, the reperfusion alone can also increase MPTP opening in control heart mitochondria.

### Effect of DCD ischemia on cardiac function, cytochrome *c* release and ROS production

**Fig 6:**
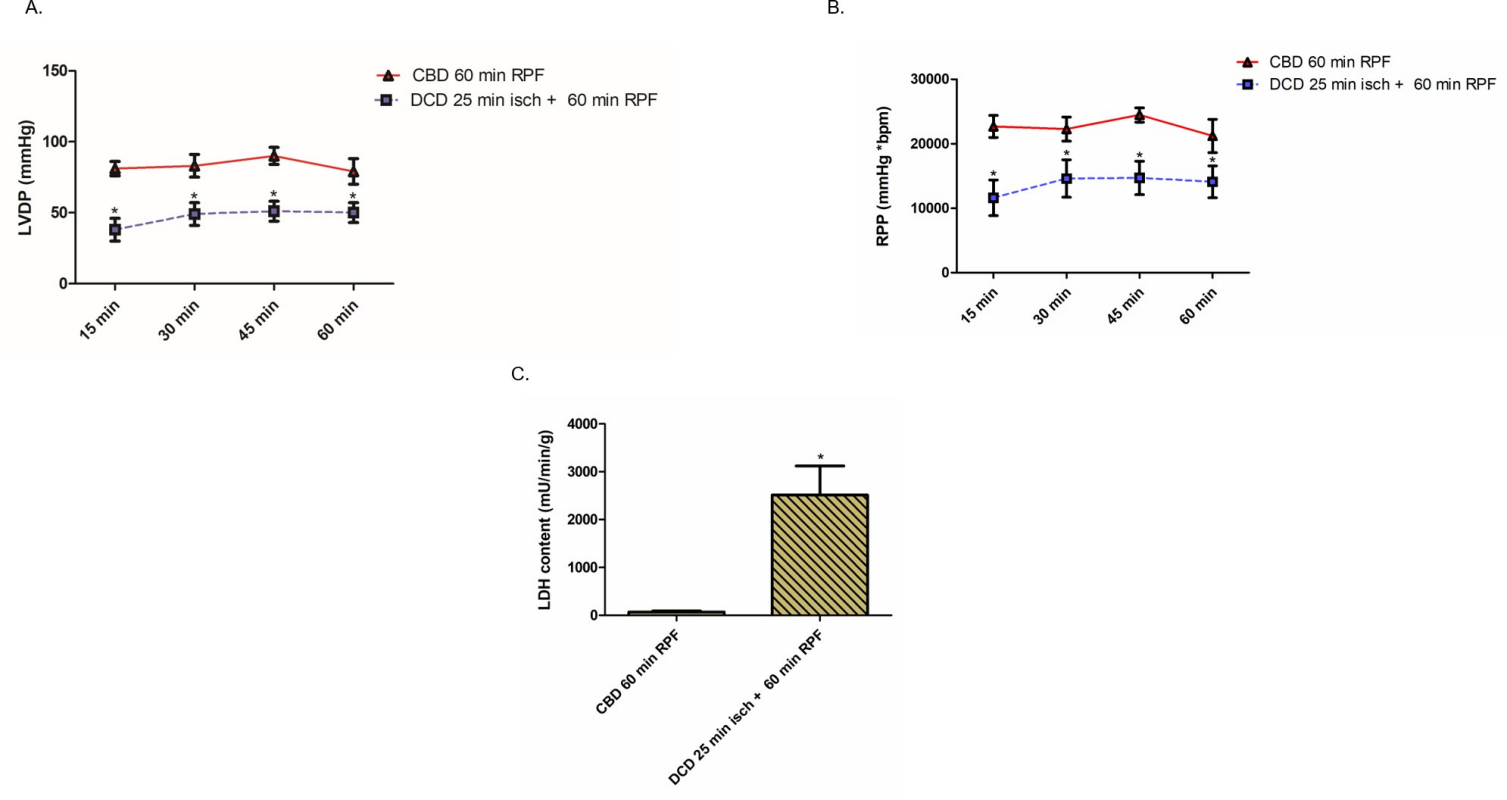
Cardiac function and lactate dehydrogenase release in CBD and DCD hearts. Heart function (left ventricle developed pressure- LVDP) or (B.) rate pressure product (RPP) was measured with a balloon tip catheter placed in the left ventricle of DCD and CBD hearts at 15, 30, 45 and 60 minutes. (C.) Timed coronary sinus samples were collected from CBD and DCD hearts (n = 8, each) to measure lactate dehydrogenase release. Data are expressed as mean ±SEM. *p<0.05 vs CBD group, using two tailed non-paired t-test.

**Fig 7:**
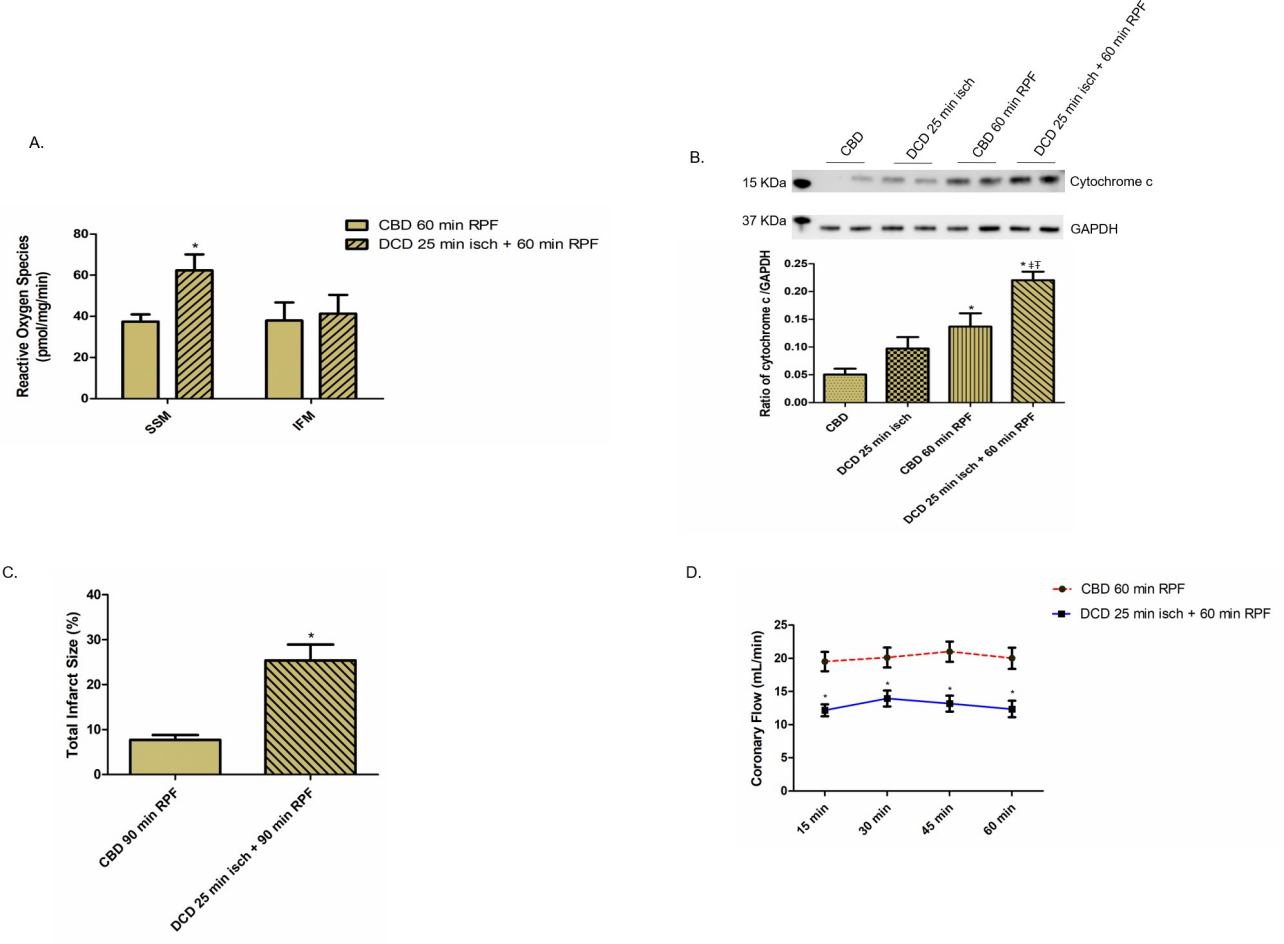
Reactive oxygen species production from mitochondria and cytosol cytochrome *c* levels in reperfused CBD and DCD hearts. (A.) H_2_O_2_ production in CBD and DCD hearts subjected to 60 minutes of reperfusion (RPF), n = 8, each. *p<0.05 vs CBD group, using two tailed non-paired t-test. (B.) Upper panel shows representative blot for immunoblotting of cytosolic cytochrome *c* with anti-cytochrome *c* antibody. Lower panel graph represents ratio of cytochrome *c* to GAPDH, the loading control. Data are expressed as mean ±SEM. *p<0.05 vs. CBD group; ^ǂ^p<0.05 vs. DCD group; ^Ŧ^p<0.05 vs. CBD with reperfusion group, using one-way ANOVA. n = 4 in each group. (C). Total infarct size as measured by triphenyl tetrazolium chloride (TTC) staining, in CBD and DCD hearts subjected to reperfusion (RPF), n = 8, each. *p<0.05 vs CBD group, using two tailed non-paired t-test. (D.) Coronary flow from CBD and DCD hearts following 60 minutes of reperfusion. *p<0.05 vs CBD group, using two tailed non-paired t-test.

Since we observed impaired mitochondrial function in DCD hearts, we elected to examine the effect on cardiac function during 60 minutes of reperfusion. We measured HR, LVDP, RPP, and positive and negative dP/dt at 15-minute intervals. There were no differences in HR (bpm) between CBD (mean ± SEM, 395 ± 11, n = 8) vs. DCD (mean ± SEM, 409 ± 9, n = 8, p = ns) hearts before stopping the ventilator. Compared to CBD hearts, DCD hearts with 25 minutes of ischemia and 60 minutes of reperfusion led to 53%, 41%, 43% and 37% decreases in LVDP measured at 15, 30, 45, and 60 minutes of reperfusion, respectively (Fig 6A). RPP, positive and negative dP/dt in the DCD group of hearts correlated with LVDP measurements compared with CBD hearts (Fig 6B and S1 Table). These findings corroborate a decrease in cardiac function in DCD hearts corresponding to impaired mitochondrial function. To further characterize cardiac injury, we determined LDH release into coronary effluent, and cytochrome *c* content in the cytosol of DCD hearts. The content of LDH in coronary effluent in DCD hearts was significantly higher compared to the CBD hearts (Fig 6C). Also, immunoblotting with anti-cytochrome *c* antibody showed a significantly increased cytochrome *c* content in the cytosol of DCD hearts compared to CBD hearts with and without reperfusion (Fig 7B). In addition, SSM from DCD hearts exhibited a profound increase (67%) in ROS production compared to CBD hearts (Fig 7A). Further, DCD hearts with 90 minutes of reperfusion exhibited increased total infarct size (as measured by triphenyl tetrazolium chloride (TTC) staining) compared to CBD hearts with 90 minutes of reperfusion (Fig 7C). Coronary flow was decreased in DCD hearts compared to CBD hearts (Fig 7D). These data support the notion that the DCD process increases ROS production from SSM, increases the release of cytosol cytochrome *c*, and also increases total infarct size.

## Discussion

HTx is limited due to the availability of heart donors, but hearts from DCD donors can expand the heart donor pool. However, DCD hearts have significant myocardial injury as shown with increased infarct size (27%, compared to 7% in CBD hearts; {Fig 7C}), in hearts following a relatively longer period of ischemia. Thus, it is, imperative to identify potential interventions that could diminish the inherent ischemia and reperfusion related damages that occur during the DCD process. This study evaluated mitochondrial and cardiac function in response to ischemia and reperfusion in rat DCD hearts. The main findings of this study are that the mitochondria from DCD hearts have; a) decreased OXPHOS, b) excess ROS production, c) increased susceptibility to MPTP opening, and that d) reperfusion further exacerbated the mitochondrial injury by enhancing MPTP opening. We noticed SSM to be more susceptible to DCD injury compared to IFM and that the mitochondrial injury correlated with decreased cardiac function.

This study explored the functional derangements in cardiac mitochondria in response to ischemia, which is inherent to the DCD process. In addition to localizing the site of injury in mitochondria with ischemia, we studied the additional impact of reperfusion. In our laboratory, we developed a rat DCD heart model that closely resembles the clinical DCD process, where *in situ* ischemia and volume overload play a significant role in damaging myocardial function. The ischemia duration we selected is based on initial studies from our group and others that reported a maximum *in situ* ischemia duration of 25 minutes for rat hearts beyond which it is less likely for hearts to recover [23–25]. We, therefore, evaluated mitochondrial function from hearts undergoing 25 minutes and 35 minutes of ischemia, to provide the critical platform to identify future interventional strategies that may extend the warm ischemia time to a DCD heart beyond 25 minutes, which is the limit set by the current clinical DCD HTx practice [26].

Studies show that myocardial ischemia causes two main types of mitochondrial injury: damage to ETC and MPTP [27, 28]. Damage to ETC increases the production of ROS that favors MPTP opening, leading to the release of cytochrome *c* from mitochondria into the cytosol leading to apoptosis and necrosis [11]. In the global ischemia model (DCD), we noted damage to the ETC as shown with a decrease in the uncoupled respiration with complex I and II substrates (Fig 2A and 2B). It is interesting to note that a further increase in ischemia duration from 25 minutes to 35 minutes did not lead to a further decrease in OXPHOS (Fig 2).The possible reasons for the lack of additional injury to the ETC with a longer duration of ischemia could be a significant loss of mitochondrial function with 25 minutes of ischemia not leaving sufficient OXPHOS function for further injury. We noticed that SSM were more susceptible to ischemic injury than IFM, which is consistent with previous observations [7, 21]. The increased susceptibility of SSM to ischemia is likely a reflection of the different tolerance to calcium-mediated injury, the regional environment of SSM, and potentially their metabolic roles [21, 29, 30]. It is interesting to speculate whether the protection of IFM alone is sufficient to mitigate cardiac injury in the DCD setting. However, with the progression of ischemic damage, SSM can release cytochrome *c* even with intact IFM [18], potentially resulting in the activation of programmed cell death.

To localize the site of injury in mitochondria that leads to a decrease in OXPHOS, we used dinitrophenol (DNP), a commonly used uncoupling agent that allows the flow of protons down the electrochemical gradient into the mitochondrial matrix bypassing complex V (the normal pathway for proton flow back into the matrix concomitant with the phosphorylation of ADP) [21] with eventual collapse of membrane potential. We observed a reduction in DNP uncoupled respiration in SSM oxidizing complex I and II substrates and in IFM oxidizing complex I but not in complex II substrates (Table 1). Thus, the decreased respiration in DCD mitochondria with uncoupler localized the damage at the ETC. There was also an increase in state 4 respiration of SSM and IFM (S2 Table), which suggests a potential permeabilization of mitochondrial membrane.

**Table 1:** 2,4 -Dinitrophenol uncoupled respiration in CBD and DCD hearts.

	CBD	DCD 25 min ischemia	DCD 35 min ischemia
	n = 5	n = 5	n = 5
**SSM**			
**Complex I substrate**			
DNP supplemented respiration- nAO/mg/min	211 ± 33	109 ± 10*	96 ± 26*
**Complex II substrate**			
DNP supplemented respiration- nAO/mg/min	198 ± 23	123 ± 4*	131 ± 24*
**IFM**			
**Complex I substrate**			
DNP supplemented respiration- nAO/mg/min	292 ± 21	217 ± 18*	150 ± 39*
**Complex II substrate**			
DNP supplemented respiration- nAO/mg/min	247 ± 55	196 ±15	151 ± 30

Our study establishes the site of injury in mitochondria from DCD related ischemia to the ETC, predominantly in complex I. This represents a potential site of targeted intervention to reduce injury from the DCD process, and it has a strong translational potential. Amobarbital, a reversible inhibitor of complex I, is known to protect complex I from ischemic injury [17]. The option of studying the effects of this agent, known to transiently inhibit complex I, to reduce the initiation of self-propagating ETC damage and release of the ROS cycle is appealing.

Besides the ETC, MPTP are a second target of ischemic damage from the DCD process. We found a significant decrease in CRC from DCD heart mitochondria compared to CBD hearts (Fig 5). In addition, the content of cytochrome *c* in the cytosol was markedly increased in DCD hearts, supporting the loss of cytochrome *c* from DCD heart mitochondria (Fig 7B). MPTP opening favors ROS generation, intracellular calcium overload and impairment of ATP production, all of which culminate in cell death [11–14]. Even though SSM tend to be more sensitive to calcium overload than IFM [29], we found a substantially increased susceptibility to MPTP opening in both SSM and IFM form DCD hearts (Fig 5A). Interestingly, MPTP opening is amenable to modulation with cyclosporine A that has been shown to protect mitochondria from reperfusion injury [31]. Identifying the role of MPTP opening in DCD heart injury provides an additional opportunity to modulate the injury in DCD hearts, protective benefits of which need to be explored with further studies. We noticed a correlation between mitochondrial injury and the physiologic heart function of DCD hearts reanimated on the Langendorff perfusion system. CBD and DCD hearts had comparable heart rate; however, the developed pressure, coronary flow, +dP/dt and–dP/dt, were decreased in DCD hearts (Figs 6A, 7D, and S1 Table). The loss of heart function also correlated with significantly elevated LDH levels in coronary flow (Fig 6C). The correlation between mitochondrial injury and decreased heart function in DCD hearts as well as increased myocyte death (indicated by LDH release, and increased infarct size {Fig 7C}) provides an opportunity to measure the effectiveness of mitochondrial protective interventions such as amobarbital and cyclosporine A, in restoring the heart function in future studies.

Reperfusion injury is well documented in regional ischemia and reperfusion models [32, 33]. We examined the contribution of reperfusion injury at the mitochondrial level in DCD hearts. Buffer perfusion of CBD hearts did not cause any decrease in mitochondrial respiratory function (Fig 3). In contrast, OXPHOS, and especially CRC were further impaired in mitochondria from DCD hearts with 60 minutes of reperfusion, supporting the notion that reperfusion following ischemia further impairs the ETC and opens the MPTP [32, 33]. Interestingly, we observed a slight improvement in OXPHOS using complex I substrate in DCD hearts with 10 min of reperfusion compared to the DCD hearts with no reperfusion. The mechanisms involved in ischemia-reperfusion mediated complex I defect includes conversion of complex I from active form to the inactive form [34], post-translational modifications [35], and subunit degradation [32]. Reperfusion with oxygenated buffer leads to normalization of intracellular pH that may improve OXPHOS with complex I substrate [36]. In addition, the regeneration of NADH during early reperfusion can improve complex I activity by switching the inactive form to its active form [34]. These factors may lead to a temporary improvement in OXPHOS during early reperfusion (as we noticed in DCD hearts reperfused for 10 minutes) however, prolonged reperfusion leads to decreased complex I activity by cleaving its subunits [32, 37].

In addition to a decrease in CRC with reperfusion in DCD heart mitochondria, we noticed a significant release of cytochrome *c* into the cytosol (Fig 7B). Our study demonstrates that the prolonged reperfusion rather than a short period of reperfusion impaired OXPHOS and sensitized to MPTP opening in DCD hearts. These results indicate that a dual window of opportunity may exist to decrease mitochondrial damage in the DCD hearts with interventions applied at both early and during the duration of reperfusion.

Although hearts from CBD do not undergo warm ischemia, protective interventions are applied to preserve heart function and decrease the chances of myocardial damage during storage and transportation. These interventions include administering cardioplegia at the time of heart procurement and restricting the cold ischemic period to four hours or less. In contrast, no pre-mortem interventions are allowed in DCD hearts due to socio-ethical reasons [38]. Since warm ischemia is inevitable with the DCD process, damage to mitochondria is already established in these hearts [19]. A recent study showed that mitochondrial transplantation decreased cardiac injury in rabbit hearts following *in vivo* ischemia-reperfusion injury by improving mitochondrial function [39]. Therefore, replacing the damaged mitochondria with mitochondrial transplantation is a potential option to improve mitochondrial function in DCD hearts. Our current study shows that the sensitivity of MPTP opening is further increased in mitochondria following reperfusion compared to ischemia. Thus, timely intervention at the onset of reperfusion with cyclosporine A may be a window of opportunity to decrease MPTP opening in DCD hearts [40]. Future studies can explore the effectiveness of this option.

### Limitations

Several mechanisms play a role in ischemia/reperfusion-induced cardiac injury; our work is a focused study of the mitochondrial disorders resulting from these events. Since we used non-blood based perfusate, the interplay of ischemia/reperfusion injury with immune-modulating cells (leukocytes) is not accounted for in our results. While blood-based perfusates are physiologic, the supply and storage of blood add to the complexity of *ex situ* perfusion. We limited reperfusion to 60 minutes for evaluation of mitochondrial function and cannot comment on additional injury beyond 60 minutes. Although this work evaluated the mitochondrial and cardiac derangements in DCD hearts, future work will examine the protective effects of amobarbital, a complex I inhibitor, and cyclosporine A, an MPTP inhibitor, in DCD hearts. In addition, mitochondrial transplantation may also be a potential strategy to improve mitochondrial function in hearts following circulatory death.

## Conclusions

Mitochondrial dysfunction is a critical consequence of ischemia in DCD hearts. Ischemia causes ETC damage, primarily at complex I, leading to ROS production and the onset of MPTP opening. These mitochondrial responses trigger mechanisms that contribute to cardiac injury, manifested as decreased heart function. For DCD hearts to be considered for transplantation, interventions targeted at protecting mitochondria before ischemia and during reperfusion are critical.

## Supporting information

S1 Table Cardiac function in CBD and DCD hearts subjected to 60 minutes of reperfusion. (DOCX)

S2 Table Mitochondrial oxidative phosphorylation in CBD and DCD hearts. (DOCX)

S3 Table Mitochondrial oxidative phosphorylation in CBD hearts with and without reperfusion. (DOCX)

S4 Table Mitochondrial oxidative phosphorylation in DCD hearts with and without reperfusion. (DOCX)

S1 Visual abstract (DOCX)

S1 Raw image Cytochrome c blot. (PDF)

S2 Raw image GAPDH control for cytochrome c blot. (PDF)

S1 Checklist ARRIVE guideline checklist. (DOCX)
