# Supplementary material for: Ischemia and reperfusion injury to mitochondria and cardiac function in donation after circulatory death hearts- an experimental study
Source: PLoS One. 2020 Dec 28;15(12):e0243504. doi: 10.1371/journal.pone.0243504 (PMC7769461; doi:10.1371/journal.pone.0243504)
Supplement: S1 Table — (DOCX) [file pone.0243504.s001.docx]

**S1 Table: Cardiac function in CBD and DCD hearts subjected to 60 minutes of reperfusion**

|  | **CBD + 60 minutes of reperfusion**  **n = 8** | | | | **DCD + 60 minutes of reperfusion**    **n = 8** | | | |
| --- | --- | --- | --- | --- | --- | --- | --- | --- |
| Value measurement time in minutes | 15 | 30 | 45 | 60 | 15 | 30 | 45 | 60 |
| Heart rate- bpm | 281 ± 16 | 273 ± 13 | 277 ± 15 | 272 ± 15 | 297 ± 14 | 290 ± 12 | 284 ± 10 | 276 ± 10 |
| +dP/dt -mmHg/s | 3113  ±  219 | 3164  ±  302 | 3365  ±  268 | 3517  ±  271 | 1639  ±  278* | 2065  ±  334* | 1967  ±  189* | 1957  ±  201* |
| -dP/dt- mmHg/s | -2563 ± 142 | -2625 ± 251 | -2818 ± 210 | -2914 ± 188 | -1622 ± 243* | -1848 ± 298* | -1701 ± 156* | -1661 ± 152* |

Table shows cardiac function measurements in CBD and DBD groups with 60 minutes of reperfusion. +dP/dt = rate of positive LVDP/second, -dP/dt = rate of negative LVDP/second. Values represent mean ± standard error of mean. *p ˂0.05 vs CBD group, using two tailed non paired student t-test.
