# Supplementary material for: Ischemia and reperfusion injury to mitochondria and cardiac function in donation after circulatory death hearts- an experimental study
Source: PLoS One. 2020 Dec 28;15(12):e0243504. doi: 10.1371/journal.pone.0243504 (PMC7769461; doi:10.1371/journal.pone.0243504)
Supplement: S2 Table — (DOCX) [file pone.0243504.s002.docx]

**S2 Table: Mitochondrial oxidative phosphorylation in CBD and DCD hearts**

|  | **CBD**  **n = 5** | **DCD 25 min ISCH**  **n = 5** | **DCD 35 min ISCH**  **n = 5** |
| --- | --- | --- | --- |
| Rat Body weight- in grams | 346 ± 13 | 292 ± 9 | 299 ± 5 |
| Rat Heart weight- in grams | 1.1 ± 0.1 | 1.2 ± 0 | 1.2 ± 0 |
| SSM Protein yield- mg/g tissue | 15.4 ± 1.3 | 12.4 ± 0.6 | 11.4 ± 1.6 |
| **SSM** | | | |
| **Complex I substrate** | | | |
| State 3 respiration**-** nAO/mg/min | 201 ± 18 | 134 ± 6.6* | 124 ± 22* |
| State 4 respiration**-** nAO/mg/min | 18 ± 3 | 29 ± 5.4 | 32 ± 9 |
| RCR | 13 ± 2.1 | 6 ± 1.4* | 5.6 ± 2.2* |
| **Complex II substrate** | | | |
| State 3 respiration**-** nAO/mg/min | 294 ± 65 | 138 ± 7* | 151 ± 29* |
| State 4 respiration**-** nAO/mg/min | 79 ± 12 | 61 ± 4.1 | 61 ± 5 |
| RCR | 3.6 ± 0.2 | 2.3 ± 0.2* | 2.5 ± 0.5 |

|  | **CBD**  **n = 5** | **DCD 25 min ISCH**  **n = 5** | **DCD 35 min ISCH**  **n = 5** |
| --- | --- | --- | --- |
| IFM Protein yield- mg/g tissue | 14.2 ± 0.8 | 12.6 ± 1.2 | 12 ± 0.6 |
| **IFM** | | | |
| **Complex I substrate** | | | |
| State 3 respiration**-** nAO/mg/min | 256 ± 16 | 221 ± 17 | 173 ± 26* |
| State 4 respiration**-** nAO/mg/min | 25 ± 2 | 39 ± 4* | 48 ± 12 |
| RCR | 11.2 ± 1.0 | 6.0 ± 1.0* | 4.8 ± 1.6* |
| **Complex II substrate** | | | |
| State 3 respiration**-** nAO/mg/min | 360 ± 79 | 228 ± 13 | 191 ± 33 |
| State 4 respiration**-** nAO/mg/min | 105 ± 25 | 89 ± 4 | 79 ± 6 |
| RCR | 3.6 ± 0.4 | 2.6 ± 0.2* | 2.5 ± 0.4 |

Table shows oxidative phosphorylation (OXPHOS) from complexes I and II in subsarcolemmal mitochondria (SSM) and interfibrillar mitochondria (IFM) from donation after circulatory death (DCD) hearts with 25 and 35 minutes of ischemia (ISCH) compared to SSM and IFM from control beating-heart donor (CBD) hearts. The unit of measurement for OXPHOS is nanoatoms of atomic oxygen/mg/min of mitochondrial protein (nAO/mg/min). RCR = respiratory control ratio. Values represent mean ± standard error of mean. *p ˂0.05 vs CBD group, using one-way ANOVA.
