# Supplementary material for: Ischemia and reperfusion injury to mitochondria and cardiac function in donation after circulatory death hearts- an experimental study
Source: PLoS One. 2020 Dec 28;15(12):e0243504. doi: 10.1371/journal.pone.0243504 (PMC7769461; doi:10.1371/journal.pone.0243504)
Supplement: S3 Table — (DOCX) [file pone.0243504.s003.docx]

**S3 Table: Mitochondrial oxidative phosphorylation in CBD hearts with and without reperfusion**

|  | **CBD**  **n = 5** | **CBD + 60 minutes of reperfusion**  **n = 8** |
| --- | --- | --- |
| Rat body weight- in grams | 346 ± 13 | 355 ± 6 |
| Heart weight- in grams | 1.1 ± 0.1 | 1.32 ± 0.1 |
| SSM protein yield- mg/g tissue | 15.4 ± 1.3 | 13.5 ± 1.0 |
| **SSM** | | |
| **Complex I substrate** | | |
| State 3 respiration**-** nAO/mg/min | 201 ± 18 | 197 ± 22 |
| State 4 respiration**-** nAO/mg/min | 18 ± 3 | 29 ± 3* |
| RCR | 13 ± 2 | 6.8 ± 0.4* |
| DNP supplemented respiration**-** nAO/mg/min | 211 ± 33 | 228 ± 33 |
| **Complex II substrate** | | |
| State 3 respiration**-** nAO/mg/min | 294 ± 65 | 189 ± 20 |
| State 4 respiration**-** nAO/mg/min | 79 ± 12 | 69 ± 6 |
| RCR | 3.6 ± 0.2 | 2.8 ± 0.3 |
| DNP supplemented respiration**-** nAO/mg/min | 198 ± 23 | 171 ± 24 |

|  | **CBD**  **n = 5** | **CBD + 60 minutes of reperfusion**  **n = 8** |
| --- | --- | --- |
| IFM protein yield- mg/g tissue | 14.2 ± 0.8 | 12.4 ± 0.9 |
| **IFM** | | |
| **Complex I substrate** | | |
| State 3 respiration**-** nAO/mg/min | 256 ± 16 | 242 ± 28 |
| State 4 respiration**-** nAO/mg/min | 25 ± 2 | 41 ± 3* |
| RCR | 11 ± 1 | 6.3 ± 0.8* |
| DNP supplemented respiration**-** nAO/mg/min | 292 ± 21 | 304 ± 43 |
| **Complex II substrate** | | |
| State 3 respiration**-** nAO/mg/min | 360 ± 79 | 277 ± 25 |
| State 4 respiration**-** nAO/mg/min | 105 ± 25 | 115 ± 6 |
| RCR | 3.6 ± 0.4 | 2.5 ± 0.2* |
| DNP supplemented respiration**-** nAO/mg/min | 247 ± 55 | 240 ± 34 |

Table shows oxidative phosphorylation (OXPHOS) from complexes I and II in subsarcolemmal mitochondria (SSM) and interfibrillar mitochondria (IFM) from control beating-heart donor (CBD) group subjected to 60 minutes of reperfusion compared to SSM and IFM from CBD group with no reperfusion. Values represent mean ± standard error of mean. RCR = respiratory control ratio, DNP = 2,4 -dinitrophenol. *p ˂0.05 vs CBD group with no reperfusion, using two tailed non paired student t-test.
