# Supplementary material for: Ischemia and reperfusion injury to mitochondria and cardiac function in donation after circulatory death hearts- an experimental study
Source: PLoS One. 2020 Dec 28;15(12):e0243504. doi: 10.1371/journal.pone.0243504 (PMC7769461; doi:10.1371/journal.pone.0243504)
Supplement: S4 Table — (DOCX) [file pone.0243504.s004.docx]

**S4 Table: Mitochondrial oxidative phosphorylation in DCD hearts with and without reperfusion**

|  | **DCD 25 min ISCH**  **n = 5** | **DCD 25 min ISCH + 10 min reperfusion**  **n = 8** | **DCD 25 min ISCH + 60 min reperfusion**  **n = 8** |
| --- | --- | --- | --- |
| Rat body weight- in grams | 292 ± 9 | 318 ± 10 | 343 ± 20 |
| Heart weight- in grams | 1.2 ± 0 | 1.2 ± 0.1 | 1.3 ± 0.1 |
| SSM protein yield- mg/g tissue | 12.4 ± 0.6 | 10.6 ± 0.8 | 10 ± 0.3 |
| **SSM** | | | |
| **Complex I substrate** | | | |
| State 3 respiration**-** nAO/mg/min | 134 ± 7 | 163 ± 13 | 123 ± 10ǂ |
| State 4 respiration**-** nAO/mg/min | 29 ± 5 | 36 ± 3 | 33 ± 2 |
| RCR | 6 ± 1.4 | 4.9 ± 0.6 | 3.8 ± 0.4 |
| DNP supplemented respiration**-** nAO/mg/min | 109 ± 10 | 171 ± 21* | 139 ± 15 |
| **Complex II substrate** | | | |
| State 3 respiration**-** nAO/mg/min | 138 ± 7 | 189 ± 13* | 147 ± 11ǂ |
| State 4 respiration**-** nAO/mg/min | 61 ± 4 | 78 ± 5* | 68 ± 4 |
| RCR | 2.3 ± 0.2 | 2.5 ± 0.2 | 2.2 ± 0.2 |
| DNP supplemented respiration**-** nAO/mg/min | 123 ± 4 | 141 ± 15 | 125 ± 13 |

|  | **DCD 25 min ISCH**  **n = 5** | **DCD 25 min ISCH + 10 min reperfusion**  **n = 8** | **DCD 25 min ISCH + 60 min reperfusion**  **n = 8** |
| --- | --- | --- | --- |
| IFM protein yield- mg/g tissue | 12.6 ± 1.2 | 11 ± 0.5 | 11.3 ± 0.7 |
| **IFM** | | | |
| **Complex I substrate** | | | |
| State 3 respiration**-** nAO/mg/min | 221 ± 17 | 227 ±13 | 181 ± 15ǂ |
| State 4 respiration**-** nAO/mg/min | 39 ± 4 | 49 ± 7 | 46 ± 3 |
| RCR | 6 ± 1.0 | 5.3 ± 1 | 4.1 ± 0.5* |
| DNP supplemented respiration**-** nAO/mg/min | 217 ± 18 | 241 ± 17 | 206 ± 18 |
| **Complex II substrate** | | | |
| State 3 respiration**-** nAO/mg/min | 228 ± 13 | 263 ± 18 | 206 ± 19ǂ |
| State 4 respiration**-** nAO/mg/min | 89 ± 4 | 116 ± 10* | 96 ± 2*ǂ |
| RCR | 2.6 ± 0.2 | 2.4 ± 0.4 | 2.2 ± 0.2 |
| DNP supplemented respiration**-** nAO/mg/min | 196 ± 15 | 202 ± 18 | 185 ± 16 |

Table shows oxidative phosphorylation (OXPHOS) from complexes I and II in subsarcolemmal mitochondria (SSM) and interfibrillar mitochondria (IFM) from DCD hearts with 25 minutes of ischemia (ISCH) subjected to 10- or 60- minutes of reperfusion compared to SSM and IFM from donation after circulatory death (DCD) hearts with 25 minutes of ischemia. RCR = respiratory control ratio, DNP = 2,4 -dinitrophenol. Values represent mean ± standard error of mean. *p ˂0.05 vs DCD 25 min isch group; ^ǂ^p ˂0.05 vs DCD 25 min isch +10 min reperfusion, using one-way ANOVA.
