# Supplementary material for: Ischemia and reperfusion injury to mitochondria and cardiac function in donation after circulatory death hearts- an experimental study
Source: PLoS One. 2020 Dec 28;15(12):e0243504. doi: 10.1371/journal.pone.0243504 (PMC7769461; doi:10.1371/journal.pone.0243504)
Supplement: S1 Visual abstract — (DOCX) [file pone.0243504.s005.docx]

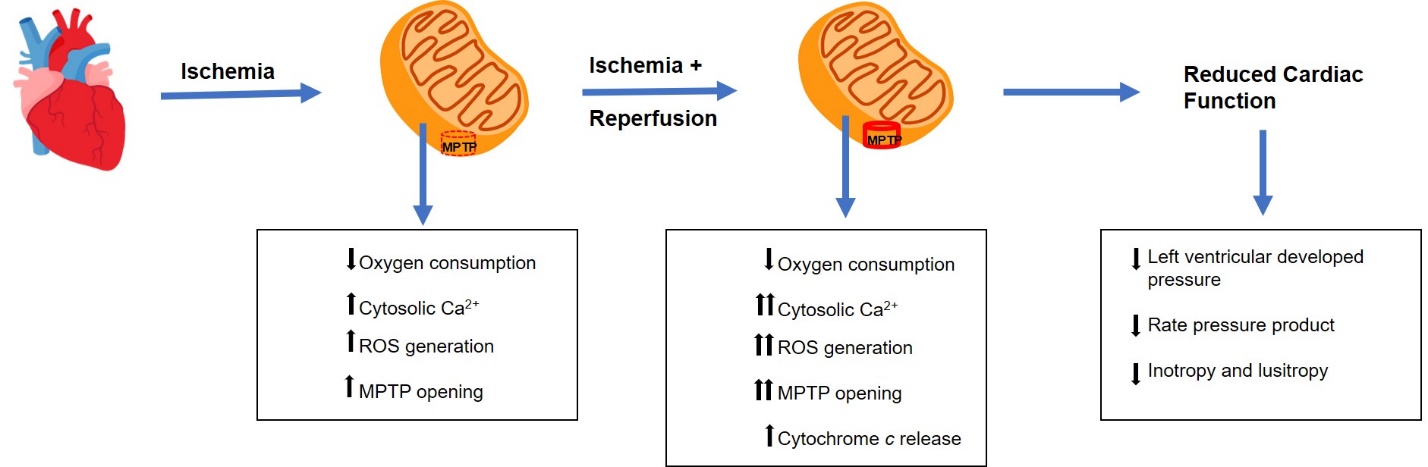


The inherent ischemia associated with the DCD process causes damage to the mitochondrial electron transport chain leading to decreased ATP synthesis, increased production of reactive oxygen species, increased intra-mitochondrial calcium, and the onset of mitochondrial permeability transition pore (MPTP) opening. Upon reperfusion, mitochondria sustain further damage and permeabilization of the mitochondria outer membrane that leads to the release of cytochrome c, an apoptosis-inducing protein. All of these processes subsequently lead to reduced cardiac function.
