# Supplementary figures and images for: Ischemia and reperfusion injury to mitochondria and cardiac function in donation after circulatory death hearts- an experimental study
Source: PLoS One. 2020 Dec 28;15(12):e0243504. doi: 10.1371/journal.pone.0243504 (PMC7769461; doi:10.1371/journal.pone.0243504)

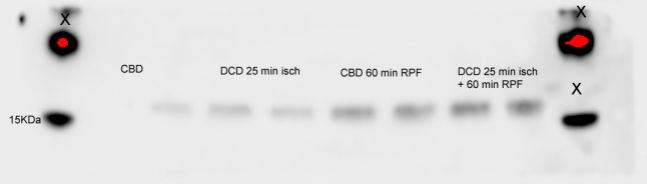

Supplement: S1 Raw image — (PDF) [file pone.0243504.s006.pdf]

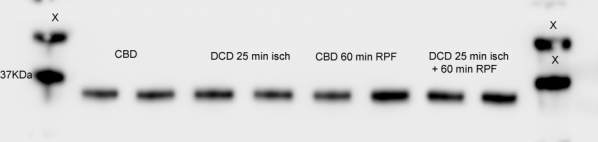

Supplement: S2 Raw image — (PDF) [file pone.0243504.s007.pdf]
