# Supplementary material for: Ischemia and reperfusion injury to mitochondria and cardiac function in donation after circulatory death hearts- an experimental study
Source: PLoS One. 2020 Dec 28;15(12):e0243504. doi: 10.1371/journal.pone.0243504 (PMC7769461; doi:10.1371/journal.pone.0243504)
Supplement: S1 Checklist — (DOCX) [file pone.0243504.s008.docx]

**Checklist for ARRIVE guidelines**

**ESSENTIAL 10**

**1. Study Design**

a. Male Sprague-Dawley rats were anesthetized with sodium pentobarbital (100 mg/kg intraperitoneally) and ventilated while monitoring heart rhythm with EKG. Heparin (1000 U/kg intraperitoneally) for anticoagulation and vecuronium (0.5 mg/ml intramuscular) to paralyze skeletal muscles were administered. Anesthesia is monitored by checking for the absence of pedal reflexes. The DCD set-up was induced by stopping ventilation and observing asystole, followed by a preset ischemia time of 25 or 35 minutes. After 5 minutes of “standoff time” hearts were procured. The control group was CBD group. CBD hearts were procured without stopping the ventilator. Where indicated, rat hearts were perfused on a Langendorff setup with modified Krebs-Henseleit (KH) buffer. Heart function was monitored during the reperfusion duration and coronary flow was collected at every 15 minutes interval.

b. This present study used heart collected form each rat at the of the ischemia period or at the end of reperfusion. For the control group, heart from each rat was collected during ventilation. Each heart was therefore considered to be an experimental unit.

**2. Sample Size**

a. Rats were divided into 6 groups. CBD without reperfusion group: 5 hearts were analyzed; CBD with 60 min of reperfusion group: 8 hearts were analyzed; DCD 25 min of ischemia group: 5 hearts were analyzed; DCD 35 min of ischemia group: 5 hearts were analyzed; DCD 25 min ischemia + 10 min reperfusion group; 8 hearts were analyzed; and DCD 25 min ischemia + 60 min reperfusion group: 8 hearts were analyzed.

b. We selected this sample size because mitochondrial and cardiac functions were evaluated for the first time in our study model and as such, the initial intention was to gather basic evidence regarding mitochondrial and cardiac derangements, for consideration when designing more complex studies.

**3. Inclusion and exclusion criteria**

a. All rats used in this study were between 8-13 weeks of age.

b. Female rats were excluded from this study.

c. The rats were divided into 6 groups: CBD without reperfusion (n = 5), CBD with 60 min of reperfusion (n = 8), DCD 25 min of ischemia (n= 5), DCD 35 min of ischemia (n = 5), DCD 25 min ischemia + 10 min reperfusion (n = 8), and DCD 25 min ischemia +60 min reperfusion (n = 8).

**4. Randomization**

a. 6 - 8 weeks-old male Sprague-Dawley rats, were obtained from Envigo, USA. Animals were quarantined for a week before being used for experiments. Animals were divided into six groups for this study.

b. Younger rats were used for experiments. Rats were weighed before each experiment to ensure weight was not greater than 370 grams. Rats were between 8 -13 weeks old at the time of use.

**5. Blinding**

Two researchers were involved as follows: The first investigator administered anesthetic procedure and performed the surgical procedure. The second investigator monitored heart function during reperfusion, and isolated mitochondria from heart tissues.

**6. Outcome measures**

a. The following parameters were assessed: heart rate before stop of ventilation; heart rate during reperfusion; left ventricular developed pressure (LVDP); +dP/dt = rate of positive LVDP/second; -dP/dt = rate of negative LVDP/second; coronary flow during reperfusion; oxidative phosphorylation (OXPHOS) from isolated mitochondria; calcium retention capacity (CRC) from isolated mitochondria, LDH release and cardiac troponin level from coronary flow; infarct size from heart slices.

b. The primary outcomes of this study are: mitochondrial injury assessed by OXPHOS and CRC; cardiac injury assessed by LVDP, +dP/dt = rate of positive LVDP/second, -dP/dt = rate of

negative LVDP/second troponin and infarct size by triphenyl tetrazolium chloride TTC staining.

**7. Statistical methods**

Comparisons between two groups were performed with a two-tailed non-paired, student t-test. Comparisons between three groups were performed using a one-way analysis of variance (ANOVA) followed by Student-Newman-Keuls analysis for multiple groups when data passed the normality test. A p-value less than 0.05 from either one-way ANOVA or student t-test was considered significant. Graphs were generated using GraphPad Prism 5 software. Outliner data was defined based upon the difference between the individual data point and the mean of the remaining data greater than twice the standard deviation.

**8. Experimental Animals**

Thirty nine male Sprague-Dawley rats were used: CBD group (n=5), DCD 25 minutes of ischemia group (n= 5), DCD 35 minutes of ischemia group (n=5), CBD with buffer perfusion (n=8), DCD 25 minutes of ischemia with 10 minutes of reperfusion (n=8), DCD 25 minutes of ischemia with 60 minutes of reperfusion (n=8). All rats were purchased from Envigo, USA.

**9. Experimental procedures**

a. **Surgical procedures:** Rats were anesthetized with sodium pentobarbital (100 mg/kg intraperitoneally) and ventilated while monitoring heart rhythm with EKG. Heparin (1000 U/kg intraperitoneally) for anticoagulation and vecuronium (0.5 mg/ml intramuscular) to paralyze skeletal muscles were administered. The DCD set-up was induced by stopping ventilation and observing asystole, followed by a preset ischemia time of 25 or 35 minutes before procuring hearts. CBD hearts were procured without stopping the ventilator. After 5 minutes of “standoff time” hearts were procured and reanimated on Langendorff system. **Langendorff perfusion:** Rat hearts were perfused on a Langendorff setup with modified Krebs-Henseleit (KH) buffer (115 mM NaCl, 4.0 mM KCl, 2.0 mM CaCl_2_, 26 mM NaHCO_3_, 1.1 mM MgSO_4_, 0.9 mM KH_2_PO_4_, and 5.5 mM glucose) oxygenated with 95% O_2_/5% CO_2_, at 37^o^C perfused at a steady 72 mmHg pressure. After 10 min of reperfusion (RP), a latex balloon tip catheter was inserted into the left ventricle to monitor left ventricle function. Left ventricle developed pressure (LVDP), myocardial contractility (+dP/dt) and myocardial relaxation (-dP/dt) were measured and calculated using Labchart software (ADInstruments Inc., Colorado Springs, CO). Rate pressure product (RPP = heart rate X LVDP) was used to account for cardiac function variability with heart rate (HR). **Isolation of Mitochondria and downstream applications:** At the end of ischemia duration or reperfusion, hearts were collected for isolation of the two cardiac mitochondrial subpopulations, subsarcolemmal mitochondria (SSM), located beneath the plasma membrane and interfibrillar mitochondria (IFM), located between the myofibrils, differ in functionality. Mitochondrial protein content was measured by the Lowry method, using bovine serum album as a standard. Oxygen consumption by mitochondria (OXPHOS) was measured using a Clark-type oxygen electrode at 30°C with glutamate (20 mM, complex I substrate), succinate (20 mM) plus 7.5 μM rotenone (complex II substrate), and TMPD (N,N,N’,N’ tetramethyl p-phenylenediamine, 1 mM)-ascorbate (10 mM, complex IV substrate) + rotenone. Calcium retention capacity (CRC) was used to assess the sensitivity of MPTP opening in the isolated mitochondria. Details of mitochondrial isolation, OXPHOS, and CRC can be found in the materials and methods section of the manuscript. Details of lactate dehydrogenase release determination, H_2_O_2_ production, immunoblotting, and infarct size measurement can also be found in the materials and methods section of the manuscript.

b. To monitor heart function, heart rate, left ventricle developed pressure (LVDP), myocardial contractility (+dP/dt) and myocardial relaxation (-dP/dt) were measured every 15 minutes during reperfusion. In addition, coronary flow was collected every 15 minutes during reperfusion. Isolated mitochondria (SSM and IFM) were assessed for OXHOS, CRC, and H_2_O_2_ production within 4 hours of isolation.

c. Rats were purchased from Envigo, USA, and were quarantined for a week at the animal facility center of the McGuire VA Medical Center. Experiments were performed at the McGuire VA Medical Center.

d. The ischemia duration we selected is based on initial studies from our group and others that reported a maximum *in situ* ischemia duration of 25 minutes for rat hearts beyond which it is less likely for hearts to recover [23-25]. We, therefore, evaluated mitochondrial function from hearts undergoing 25 minutes and 35 minutes of ischemia, to provide the critical platform to identify future interventional strategies that may extend the warm ischemia time to a DCD heart beyond 25 minutes, which is the limit set by the current clinical DCD HTx practice

**10. Results**

The main findings of this study are that the mitochondria from DCD hearts have; a) decreased OXPHOS, b) excess ROS production, c) increased susceptibility to MPTP opening, and that d) reperfusion further exacerbated the mitochondrial injury by enhancing MPTP opening. All data presented in this study are expressed as means ±SEM. Where indicated, values significantly different, compared to the appropriate control group have a p value less than 0.05.

**RECOMMENDED SET**

**11. Abstract**

Donation after circulatory death (DCD) donor hearts can increase the heart donor pool. However, ischemia and reperfusion injuries associated with the DCD process causes myocardial damage, limiting the use of DCD hearts in transplantation. Addressing this problem is critical in the exploration of DCD hearts as suitable donor hearts for transplantation. In this study, sprague-dawley male rat hearts were procured following the control beating-heart donor (CBD) or DCD donation process. Changes in mitochondria and cardiac function from DCD hearts subjected to 25 or 35 minutes of ischemia followed by 60 minutes of reperfusion were compared to CBD hearts. Following ischemia, rates of oxidative phosphorylation and calcium retention capacity were progressively impaired in DCD hearts compared to CBD hearts. Reperfusion caused additional mitochondrial dysfunction in DCD hearts. Developed pressure, inotropy and lusitropy, were significantly reduced in DCD hearts compared to CBD hearts. We, therefore, suggest that interventional strategies targeted before the onset of ischemia and at reperfusion could protect mitochondria, thus potentially making DCD hearts suitable for heart transplantation.

**12. Background**

a. Presently, most transplantable hearts come from donation after brain death (DBD) donors [3]. However, the availability of these donors is limited. Donation after circulatory death (DCD) donors can expand the heart donor pool. DCD hearts are not routinely used for HTx mainly because of the inherent ischemic insult resulting from the DCD process. In contrast to DBD donors that have intact cardiorespiratory function before procurement, DCD heart donors suffer from warm ischemia, which is the interval from the withdrawal of mechanical ventilation to initiation of coronary perfusion with cold organ preservation solution**.** In addition, during the DCD process, the heart experiences a rapid surge of catecholamines and volume overload that also contribute to the myocardial damage.

Mitochondria are key targets of myocardial injury during ischemia and reperfusion. Mitochondrial function is known to be impaired in DCD organs such as kidney and liver. Most of the knowledge on the series of events and on the extent of damage to the mitochondria in the ischemic or reperfused hearts comes from *ex-vivo* ischemia studies. In this study we will address the changes in mitochondrial and cardiac function from DCD hearts subjected to different periods of *in vivo* ischemia and *in vitro* reperfusion.

b. In this study, we used young male Sprauge-Dawley rats (8-13 weeks old), an outbred multipurpose breed of albino rats used extensively in medical research. Its main advantage is its calmness and ease of handling. In our laboratory, we developed a rat DCD heart model that closely resembles the clinical DCD process, where *in situ* ischemia and volume overload play a significant role in damaging myocardial function. The ischemia duration we selected is based on initial studies from our group and others that reported a maximum *in situ* ischemia duration of 25 minutes for rat hearts beyond which it is less likely for hearts to recover [23-25]. We, therefore, evaluated mitochondrial function from hearts undergoing 25 minutes and 35 minutes of ischemia, to provide the critical platform to identify future interventional strategies that may extend the warm ischemia time to a DCD heart beyond 25 minutes, which is the limit set by the current clinical DCD HTx practice.

**13. Objectives**

The primary objective of this study was to investigate the changes in mitochondrial and cardiac function from DCD hearts subjected to different periods of *in vivo* ischemia and *in vitro* reperfusion.

**14. Ethical Statement**

All experiments were conducted per the ‘Guide for the care and use of laboratory animals’ published by the National Institutes of Health. The Animal Care and Use Committees of the McGuire VA Medical Center and Virginia Commonwealth University (VCU) approved the present study.

**15. Housing and Husbandry**

The Division of Animal Resources at McGuire VA Medical Center and Virginia Commonwealth University are under the supervision of full-time veterinarians. Rats are allowed to equilibrate for at least a week following arrival before use. The maximum caging density was two rats. Rats were fed with standardized rat diet and provided drinking water ad libitum. VCU and McGuire VA Medical Center animal facilities are in compliance with all applicable federal, state, and local laws and university polices.

**16. Animal care and monitoring**

a. Experiments on animals were conducted in accordance with the principles outlined in the NIH Guide for the Care and Use of Laboratory Animals and conformed to the PHS policy on Humane Care and Use of Laboratory Animals. Rats were anesthetized with sodium pentobarbital (100 mg/kg intraperitoneally) and ventilated while monitoring heart rhythm with EKG. Heparin (1000 U/kg intraperitoneally) for anticoagulation and vecuronium (0.5 mg/ml intramuscular) to paralyze skeletal muscles were administered. Anesthesia was monitored by checking for the absence of pedal reflexes. Only personnel having received approval from the Institutional Animal Care and Use Committee (IACUC) of Virginia Commonwealth university and McGuire VA Medical Center participated in experiments with rats.

b. The veterinary staff monitored the overall welfare of the rats. Health was monitored by weight, food and water intake, and general assessment of animal activity, panting, and fur condition. The research team measured rat weight before each experiment to ensure weight range for the study.

**17. Interpretation/scientific implications**

a. In summary, mitochondrial dysfunction is a critical consequence of ischemia in DCD hearts. Our data show that ischemia causes ETC damage, primarily at complex I, leading to ROS production and the onset of MPTP opening. These mitochondrial responses trigger mechanisms that contribute to cardiac injury, manifested as decreased heart function. For DCD hearts to be considered for transplantation, interventions targeted at protecting mitochondria before ischemia and during reperfusion are critical.

b. Several mechanisms play a role in ischemia/reperfusion-induced cardiac injury; our work is a focused study of the mitochondrial disorders resulting from these events. Since we used non-blood based perfusate, the interplay of ischemia/reperfusion injury with immune-modulating cells (leukocytes) is not accounted for in our results. While blood-based perfusates are physiologic, the supply and storage of blood add to the complexity of *ex situ* perfusion. We limited reperfusion to 60 minutes for evaluation of mitochondrial function and cannot comment on additional injury beyond 60 minutes. Although this work evaluated the mitochondrial and cardiac derangements in DCD hearts, future work will examine the protective effects of amobarbital, a complex I inhibitor, and cyclosporine A, an MPTP inhibitor, in DCD hearts. In addition, mitochondrial transplantation may also be a potential strategy to improve mitochondrial function in hearts following circulatory death.

**18. Generalizability/translation**

From this study, the correlation between mitochondrial injury and decreased heart function in DCD hearts, as well as increased myocyte death (indicated by LDH release, and increased infarct size provides an opportunity to measure the effectiveness of mitochondrial protective interventions such as amobarbital and cyclosporine A, in restoring the heart function. These results indicate that a dual window of opportunity may exist to decrease mitochondrial damage in the DCD hearts with interventions applied at both early and during the duration of reperfusion.

**19. Protocol registration**

All protocols for animal experimentation were prepared before the study and submitted for approval to the VCU and McGuire VA Medical Center IACUC.

**20. Data access**

All relevant data are included in the main manuscript and additional data are supplied as supporting information. All data are fully available without restriction.

**21. Declaration of interests**

a. The authors have declared that no competing interests exist.

b. This work was supported by Merit Review Grant awarded to Dr. Mohammed Quader (1I01 BX003859), Dr. Edward J. Lesnefsky (2IO1 BX001355), and funds from the Pauley Heart Center to Drs. Mohammed Quader, Qun Chen and Stefano Toldo.
